# Coronary mra angiography at 3T: fat suppression versus water-fat separation

**DOI:** 10.1186/1532-429X-16-S1-P175

**Published:** 2014-01-16

**Authors:** Maryam Nezafat, Markus Henningsson, David P Ripley, Nathalie Dedieu, Gerald F Greil, John P Greenwood, Peter Börnert, Sven Plein, Rene Botnar

**Affiliations:** 1 Division of Imaging Sciences & Biomedical Engineering, Kings college London, London, UK; 2 Multidisciplinary Cardiovascular Research Centre (MCRC) & Leeds Institute of Genetics, Health and Therapeutics (LIGHT), University of Leeds, Leeds, UK; 3 Philips Research, Hamburg, Germany

## Background

Suppression of lipid signal is a basic requirement in coronary magnetic resonance angiography (CMRA) because coronary arteries are embedded in epicardial fat and signal from fat can decrease coronary vessel conspicuity. Most CMRA scans are currently performed with fat suppression techniques such as Spectral Presaturation with Inversion Recovery (SPIR). However, methods based on spectrally-selective fat saturation are sensitive to B0 and B1 field inhomogeneities. Recent improvements in chemical shift based water fat separation methods such as Dixon[1,2]provides an alternative to conventional spectrally-selective fat suppression techniques. The purpose of this study was to compare SPIR technique and Dixon water fat separation at 3.0 T for CMRA.

## Methods

This work was performed on a 3T scanner (Achieva, Philips Healthcare, Best, The Netherland) equipped with a 32-element cardiac receiver coil. Data were acquired in eight healthy volunteers (six male, two female, mean age 36 ± 11) and eight patients with suspected coronary artery disease (five male, three female, mean age 60 ± 12). Two different scans were performed in each of them: 1) Conventional whole heart CMRA with SPIR fat suppression and 2) two-point Dixon CMRA. Two experts readers, blinded to the methods used, scored the image quality for each dataset. In addition, signal-to-noise ratio of blood, fat and myocardium, contrast-to-noise ratio (CNR) between blood, fat and myocardium, and right coronary artery sharpness and length were measured to compare these two techniques quantitatively. A Wilcoxon Signed-Rank was used for statistical analysis for comparison between images acquired with Dixon and SPIR.

## Results

**Figure 1:**
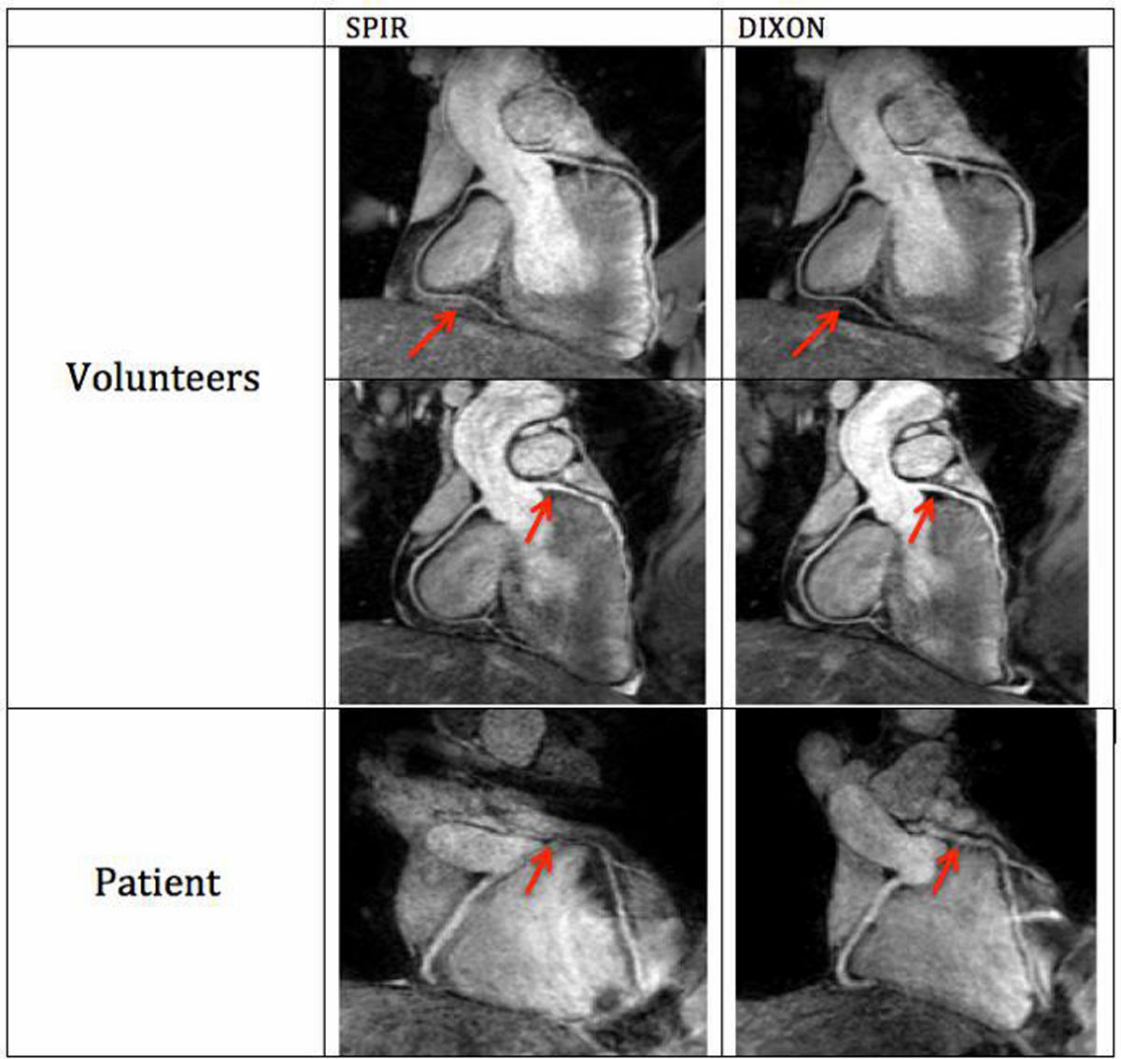
**reformatted whole heart CMRA of Dixon (first column) and SPIR (second column) acquisition data of two selected volunteers and two patients**. The arrows are pointing to locations in the images where Dixon method has performed favorably in comparison to SPIR.

**Figure 2:**
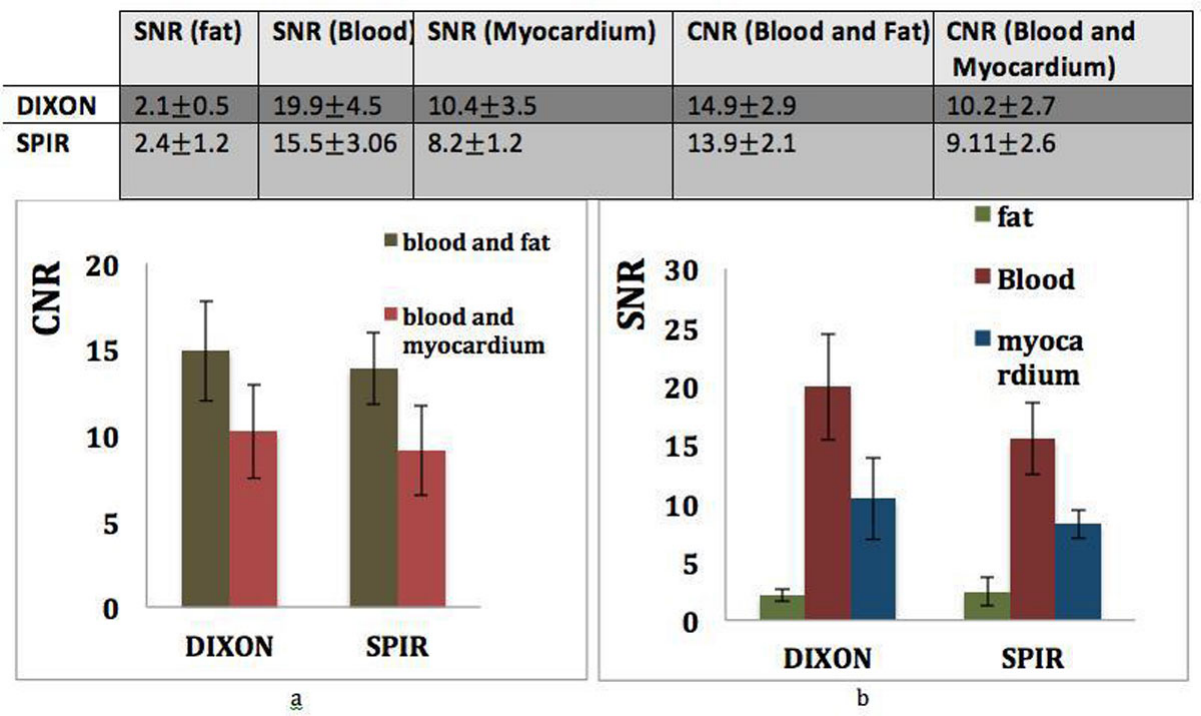
**a) SNR of blood and myocardium increase with the DIXON method in healthy subjects leading to better contrast to noise ratio (b) between blood, myocardium and fat**.

All scans were successfully performed and produced good quality images in all volunteers. Figure 1 shows representative CMRA images from two healthy volunteers and one patient acquired with SPIR and Dixon. In both volunteers fat suppression with Dixon was visually superior to the SPIR technique. Images acquired with the two-point Dixon method were scored higher than the ones acquired with SPIR in volunteers (4.4 ± 0.7 vs. 3.6 ± 0.6, p = 0.009) and in patients (2.35 ± 0.9 vs. 1.8 ± 1.2, p = 0.04). Vessel sharpness of the right coronary artery with Dixon acquisition (57 ± 0.1) was similar to SPIR (56 ± 0.1). Figure 2 demonstrates that the Dixon method leads to similar fat suppression but increased SNR and CNR compared to SPIR.

## Conclusions

These findings demonstrate that Dixon water-fat separation leads to higher SNR of coronary blood and myocardium and improved image quality scores for coronary artery visualization at high field strengths. Furthermore, the additional fat data that is available with Dixon protocols may be an important biomarker and improve the diagnostic value of CMRA.

## Funding

Wellcome Trust and ESPRC Medical Engineering Centre.
